# Tracking metal presence in cannabis vaping products from source to inhalation

**DOI:** 10.1038/s41598-025-17004-2

**Published:** 2025-08-29

**Authors:** Zuzana Gajdosechova, Joshua Marleau-Gillette, Matthew Polivchuk, Ivana Kosarac, Guru Prasad Katuri, Dharani Das, Ashley Cabecinha, Andrew Waye, Hanan Abramovici

**Affiliations:** 1 National Research Council Canada, Metrology, 1200 Montreal Rd., Ottawa, ON K1A 0R6 Canada; 2 Natural Resource Canada, 601 Booth St., Ottawa, ON K1A 0E8 Canada; 3 Tobacco Control Directorate, Controlled Substances and Cannabis Branch, Health Canada, 150 Tunney’s Pasture Driveway, Ottawa, K1A 0K9 ON Canada; 4 Office of Cannabis Science and Surveillance, Controlled Substances and Cannabis Branch, Health Canada, 150 Tunney’s Pasture Driveway, Ottawa, ON K1A 0K9 Canada

**Keywords:** Cannabis vape liquids, Metal nanoparticles, Single particle ICP-MS, Aerosol characterization, Heavy metals, Vape cartridge contamination, Trace element analysis, Public health exposure, Risk factors, Chemistry, Nanoscience and technology

## Abstract

Vaping cannabis liquids is a convenient method of cannabis consumption, and is considered to be a less harmful alternative to smoking cannabis. However, vaping cannabis carries its own health risks, with many uncertainties, especially concerning the presence of metal particles in vape liquids. The heterogeneous distribution of these particles within the liquid matrix poses analytical challenges in measurement reproducibility. In this study, total metals analysis was performed on five samples from six different legal Canadian cannabis vape liquid products. The results indicated that metals from the vaping device components, cobalt (Co), nickel (Ni), and zinc (Zn) significantly contributed to within-batch variability. In contrast, all analyzed metals showed significant variability among tested products. Single-particle ICP-MS detected metal particles of aluminum (Al), Co, chromium (Cr), copper (Cu), Ni, tin (Sn), and Zn in the vape liquids. To assess potential consumer exposure, cannabis cartridges were vaped using a vaping machine and the resulting aerosol was analyzed. Although the number of detected emitted particles was below the limit of quantitation, all samples produced aerosols containing metal particles of Co, Cr, Ni, lead (Pb), Sn, and Zn. Further analysis using SEM-EDS on the emptied cartridges showed cracking on the connector pin of an unused device, suggesting a potential source of contamination during use and storage. The elemental composition of the metal components in the cartridges matched the detected particles, providing strong evidence that cannabis vape liquids are contaminated by the metal components of vaping devices.

**Supplementary Information:**

The online version contains supplementary material available at 10.1038/s41598-025-17004-2.

## Introduction

Cannabis vape cartridges are electronic devices designed to produce an aerosol for vaping cannabis. The aerosol is generated by heating a resistance coil with a battery, causing the cannabis vape liquid to volatilize for inhalation. Unlike traditional cannabis smoking, which involves combustion, cannabis vape devices allow for prolonged use, are portable, discrete, and offer temperature control. They also do not produce strongly odorous smoke, and added flavors can enhance the taste of the aerosol. Additionally, vaping is considered a less harmful alternative to smoking because it does not involve combustion and generation of polycyclic aromatic hydrocarbons, which are known carcinogens and mutagens, among other harmful chemicals. These factors make vaping cannabis liquids a convenient and appealing method of cannabis consumption, contributing to the growing popularity of cannabis vaping^1,2^.

However, despite being considered less harmful than smoking, vaping still poses health risks, some of which are still being discovered. The cannabis plant is a known hyperaccumulator of metals^3^, leading to regulatory limits on metal contaminants in many jurisdictions where cannabis production is regulated. For example, in Canada, the *Cannabis Regulations* require cannabis products to meet the generally accepted tolerance limits for chemical contaminants based on Pharmacopoeia standards^4^. For cannabis vape liquids, these reference standards set maximum permitted levels (permitted daily exposures) for 24 elemental impurities^5^. In establishing these levels, inhalation studies using soluble salts (when available) were preferred over studies using particulates for inhalation safety assessment and derivation of the inhalation permitted daily exposures^5^; however, evidence suggests that cannabis vape liquids can also contain metals in particulate form^6,7^. Although substantial studies on human or animal exposures to vaped cannabis aerosols are lacking, evidence available to date on inhaled metal nanoparticles (NPs) shows they can penetrate deep into the lungs, triggering respiratory inflammation, and due to their high reactivity, may also affect extrapulmonary organs^8,9^.

Metal contamination in vape liquids, whether cannabis- or nicotine-based, is generally assumed to occur during manufacturing, which is why it is important to test products before sale and first use or activation. However, recent studies have shown that metals can also leach into nicotine vape liquids during operation^10,11^, providing clear evidence that contact with metal components in the device during use can contribute to contamination. Unfortunately, information regarding the metal composition of the individual device components is not disclosed publicly and can be difficult or impossible to find, making it challenging to trace the sources of metal contaminants in vape liquids without an analysis of the elemental composition of the device components. While some studies have investigated the elemental composition of vape devices^12–15^ or reported signs of corrosion^16^, the rapid evolution of vape device designs makes it challenging to generalize these findings.

The presence of metal contaminants in vape liquids raises potential health concerns. However, contamination alone does not necessarily imply exposure, as metals may not be transported into the aerosol which is inhaled. Nonetheless, experiments with nicotine vape pens have demonstrated the presence of metal contaminants in aerosol, in both ionic^17^ and particulate^16^ forms. While these studies have limitations and may not always be directly comparable, they indicate that consumers are indeed exposed to metals through inhalation when vaping nicotine products. Similar studies have yet to be conducted on cannabis vape liquids. To date, only a few publications have investigated metal contaminants in cannabis products^6,7,18,19^, reporting metals typically associated with resistance wires and other metal components of vaping devices. Poor reproducibility between repeated samples of the same product has been observed in these studies, which could be due to the heterogeneous distribution of metal NPs in the vape liquid. A recent publication using an analytical method for detecting metal NPs in cannabis vape liquids confirmed that these products can contain significant number of metal NPs^7^.

This publication examines the variability in metal content among cannabis vape cartridges within the same production lot, aiming to assess how representative the analysis of individual cartridges is for the entire batch. To date, no studies have evaluated both within-batch and between-product variations, an important gap with implications for regulatory agencies and the development of robust sampling protocols. Additionally, the presence and composition of metal NPs in cannabis vape liquids and their resulting aerosols are analyzed using single-particle inductively coupled plasma mass spectrometry (sp-ICP-MS). Existing data on aerosol composition remains limited, and current collection protocols present significant challenges. Here, we introduce a simplified aerosol collection method using a cannabis matrix-compatible solvent. To help identify the source of detected metal NPs, components of cannabis vape devices are also analyzed for their elemental composition using scanning electron microscopy coupled with energy-dispersive X-ray spectroscopy (SEM-EDS).

## Materials and methods

### Chemicals and reagents

Analytical grade nitric acid (HNO_3_) (J. T. Baker, VWR) was purified in-house by sub-boiling distillation using a high purity quartz still (duoPUR, Milesone) in a class 100 clean room and used for all aqueous sample and standard preparations. Elemental stock standards (1−100 ng g^− 1^) from Inorganic Ventures were obtained from Delta Scientific (Canada) and contained V, Cr, Mn, Co, Ni, Cu, Zn, As, Se, Mo, Cd, Hg, Tl, Pb, Ru, Rh, Pd, Sn, Sb, Te, Hf, Ir, Pt, and Au, which were traceable to NIST- standard reference material (SRM). Conostan S-21 multi-elemental standard (100 mg kg^− 1^), PremiSolv solvent, and HU-1 (used oil) reference material were obtained from SCP Science (Canada). Dodecanethiol and propylene glycol monomethyl ether (PGME) were obtained from Millipore-Sigma (Canada), and toluene and trichloromethane were obtained from Fisher Scientific (Canada). Citrate-stabilized 60 nm silver (Ag) nanoparticles (BioPuire™, 59 ± 6 nm, 1 mg mL^− 1^) and polystyrene coated 100 nm gold (Au) NPs (NanoXact™, 95 ± 13 nm, 1 mg mL^− 1^) in toluene were obtained from NanoComposix (USA). A standard solution of Au (1000 mg kg^− 1^) in hydrocarbon oil was obtained from LGC (UK). NIST SRM 1634c (trace elements in fuel oil) was obtained from National Institute of Standards and Technology (USA). Fish protein certified reference material (NRC DORM-5 CRM) was obtained from the National Research Council Canada. Cannabis samples were purchased from the Ontario Cannabis Store (www.ocs.ca) and available information on THC/CBD content, method of extraction, and additives can be found in Table 1. Deionized water (> 18 MΩ cm Milli-Q Element, Millipore) was used in all experiments.

**Table 1:** Manufacturer provided information for purchased cannabis vape liquid products. Mass fractions of THC and CBD compounds are reported in Mg g^−1^.

Sample ID	Packaging date	Amount (g)	THC	Total THC	CBD	Total CBD	Extract type	Ingredients
A	17-Jul-22	1	838.6	838.6	< 2.5	< 2.5	CO_2_	Cannabis extract, botanical terpenes
B	14-Nov-22	1	800	800	0	0	CO_2_	Cannabis distillate, botanical terpenes
C	15-Nov-22	0.95	835	835	1.9	2.2	EtOH	Cannabis distillate, botanical terpenes
D	15-Aug-22	0.4	783.61	783.61	< 5.00	< 5.00	EtOH	THC distillate, flavouring agents
E	29-Nov-22	1	860	860	2	2	CO_2_	Cannabis distillate, natural flavouring
F	27-Jul-22	1	847.3	847.3	3.4	3.4	CO_2_	Cannabis extract, terpenes, natural flavours

### Cannabis vape liquid collection

Using two separate vice grips, the mouthpiece of the vape cartridge was carefully removed from the body. The remainder of the cartridge, containing the cannabis vape liquid, was inverted and placed in a disposable 5 mL pipette tip, with the tip positioned within a 2 mL Eppendorf vial. This entire assembly was secured inside a 50 mL centrifuge tube and centrifuged at 4000 g for 5 min. Due to variations in the viscosity of the cannabis vape liquids, cartridges were inspected after each 5 min cycle to determine if additional cycles were required. Once most or all of the liquid had been collected in the Eppendorf vial, the vial was sealed and stored at room temperature until analysis.

### Total metals analysis

The total mass fraction was measured and compared in six different product sets (Sample A to F). Each product set contained five cartridges of the same production lot that were sampled (called sample set for comparison purposes) and each cartridge sample was sub-sampled 3 times (sample replicates).

Prior to sub-sampling, the cannabis vape liquid was heated in a heat block (Reacti-Therm Heating module, ThermoFisher) at 120 °C for 60 min and stirred using a magnetic stirrer bar. An aliquot of approximately 0.1 g was accurately weighed into microwave digestion vessels and digested in 1 mL of concentrated HCl and 5 mL of concentrated HNO_3_ using a closed vessel microwave digestion system (Multiwave 7000, Anthon Parr). The digestion program consisted of a 15 min ramp to 200 °C, followed by a 15 min hold at 200 °C. After digestion, the samples were transferred into 50 mL vials, evaporated to near dryness in a hot block, and reconstituted in 3% HCl and 2% HNO_3_. Two certified reference materials (NIST SRM 1634c and NRC DORM-5) and one method blank were included in each digestion cycle for quality control. Method blanks were carried through the entire sample preparation process to monitor for possible contamination. Each sample and CRM were prepared in triplicate.

All samples were analyzed using ICP-MS (Agilent 8900, Agilent Technologies, Santa Clara, CA, USA) equipped with a standard sample introduction system consisting of a MicroMist glass concentric nebulizer, quartz spray chamber, and quartz torch with 2.5 mm ID injector. The introduced aerosol was diluted 25-fold using an ultra-high matrix introduction (UHMI) feature. The interface was fitted with a nickel-plated copper sampling cone and a nickel skimmer cone. The instrument was operated in MS/MS mode using He collision gas (5.0 mL min^− 1^) for the detection of ^59^Co, ^52^Cr, ^63^Cu, ^55^Mn, ^118^Sn, and ^66^Zn. Additionally, oxygen gas (22%) was used as a reaction gas for detecting ^27^Al (mass shift to *m/z* 43), ^75^As (mass shift to *m/z* 91), ^111^Cd (on mass), ^56^Fe (on mass), ^23^Na (on mass), ^60^Ni (on mass), ^51^V (mass shift to *m/z* 67), and ^208^Pb (on mass). Daily optimization was performed to ensure suitable sensitivity and stability, maintaining cerium oxide ratios ˂ 1% and doubly charged ions (^70^Ce^+^ = ^140^Ce^2+^) ˂ 2%. An online internal standard of iridium (50 µL L^− 1^) was continuously mixed with calibration standards and samples to correct for instrument drift and matrix effects by calculating the ratio between the *m/*z of the element and the internal standard (*m/z* 193 for Ir). Quantitation was performed using the external calibration method, with low- and medium-concentration quality control (QC) samples, as well as a blank (2% HNO_3_), analyzed every ten samples. Sample introduction was automated using an Agilent SPS 4 autosampler with a protective cover (Agilent Technologies).

Limits of detection (LOD) were determined by analyzing nine method blanks and calculating the standard deviation (*σ*) of their elemental concentrations. The LOD was defined as three times the standard deviation (3*σ*), and the method detection limit (MDL) was calculated as the LOD multiplied by the nominal dilution factor of 70.

### Aerosol generation

Aerosol was generated using a CETI-8, eight port vaping machine (Cerulean, USA), equipped with automatic button activator. Most of the vape pens (vape cartridge connected to a battery for operation) were connected to the vaping machine using standard 7.50–10.00 mm mouthpiece adaptors. Custom adaptors were 3D printed and customized in the laboratory to ensure an appropriate seal around the mouthpiece for the samples requiring non-standard mouthpiece adaptors. Vape cartridges were kept upright for at least 24 h before vaping to allow the viscous liquid to cover the maximum contact surface of the heating coil. The ISO 20768:2018 vaping regime was followed to generate the aerosols and consisted of the following puffing parameters: puff duration = 3 s, puff volume = 55 mL, and puff interval (time delay between puffs) = 30 s. Products were vaped at a 45° angle. Accurate puff volume was verified using calibrated soap bubble meters (Cerulean, USA). Each sample was used to generate 30 or 50 puffs per puffing session. Puffing sessions for certain samples, where sample volume allowed, were repeated 3 or 5 times for a total of 150 puffs for 510-thread cartridges or 250 puffs for closed-pod cartridges, with a 10 min rest period between puffing sessions. For each experiment, products were vaped until the majority of sample and/or sufficient concentrate had been consumed and the aerosol was collected using two impingers connected in tandem, each containing 30 mL of PremiSolv. The outflow of the second impinger was connected to a HEPA filter. All aerosol capture experiments were performed in triplicate except sample A. Air blanks were obtained by setting up the same experimental collection system without connecting a vape pen and drawing in ambient air during the vaping process. After aerosol was collected, all pieces of connector tubing, the mouth piece and impingers were step-wise rinsed thoroughly with MilliQ water, methanol, and hexane. A conditioning puffing regime was carried out before each puffing session in order to prime and activate the devices. The batteries of the vaping devices were always fully charged (100%) before starting a puffing regime.

### Metal particles analysis

Metal particle analysis was conducted using an Agilent 8900 ICP-MS (Agilent Technologies, Santa Clara, CA, USA) in single-particle mode, following a previously described method^7^. Briefly, the interface was equipped with a platinum sampling and skimmer cone, a MiraMist concentric nebulizer, a quartz spray chamber, and a quartz torch with a 1 mm injector and solvent resistant tubing (SolvaFlex). Analyses were performed in time-resolved analysis (fast TRA) mode with a dwell time of 0.1 ms per point and no settling time between measurements. Daily optimization was performed using a custom-made tune solution containing Al, V, and Pb (10 µg kg^− 1^) to ensure suitable sensitivity. The instrument was operated in MS/MS mode using NH_3_ gas (3.0 mL min^− 1^, 30%) to detect ^52^Cr, ^59^Co, ^63^Cu, ^66^Zn, ^103^Ag, ^118^Sn, ^197^Au, and ^206^Pb, all detected on mass. Oxygen gas (0.38 mL min^− 1^, 25%) was used as a reaction gas for the detection of ^27^Al (on mass), ^51^V (mass shift to *m/z* 67), and ^60^Ni (on mass). Raw data were exported and processed using SPCal software (version 1.3.2) and the threshold detection was calculated using Formula C.

To determine transport efficiency, a portion of the citrate-stabilized 60 nm Ag NPs reference material was diluted to a Ag concentration of approximately 5 µg kg^− 1^ via PGME into PremiSolv. An Agilent SPS 4 autosampler with a protective cover was used for sample introduction, and a blank sample was run after each cannabis vape sample.

### Scanning electron microscopy analysis

Scanning electron microscope (SEM) imaging and chemical analysis via energy dispersive X-ray spectroscopy (EDS) were performed at the Geological Survey of Canada’s Microbeam Laboratory using a Zeiss EVO 50 SEM equipped with an Oxford Instruments X-Max 150 silicon drift X-ray detector. Uncoated samples were analyzed in variable pressure mode at a chamber pressure of 10 Pa. Analytical conditions included an accelerating voltage of 20 kV, a probe current of 750 pA, and a working distance of 8.5 mm. X-ray mapping was conducted using a pixel dwell time of 5 ms. Spot EDS analyses were performed on features of interest based on the X-ray map results using an analysis time of 3 s.

## Results and discussion

### Total metals analysis

The mass fractions of the measured Class 1 contaminants (As, Cd, and Pb) in the studied cannabis vape liquids were within the Pharmacopoeia tolerance limits^5^. The highest As mass fraction (17.6 ± 20.4 µg kg^− 1^, Table S1) was observed in a single sample, while the median mass fractions were generally below 5 µg kg^− 1^ (Table 2). Cadmium was detected above the method’s LOD (0.49 µg kg^− 1^) in only half of the studied samples, with the highest mass fraction reaching 3.20 µg kg^− 1^ (Table S1). Lead was detected in one full set of samples (Sample D) but remained below the LOD in several cartridges within each set or across entire sets. The highest Pb mass fraction measured was 69.3 ± 6.2 µg kg^− 1^ (Sample D, Table S1), which is consistent with previously reported values in cannabis vape liquids^6,18^. Regarding variability within products from the same production lot, As and Cd showed a narrow-measured range. However, assessing Pb variability was not possible due to the large number of samples with Pb mass fractions below the method’s LOD (5.5 µg kg^− 1^).

**Table 2:** Median (interquartile range) of metal mass fractions (µg kg^− 1^) in cannabis vape liquids.

Element	Sample A	Sample B	Sample C	Sample D	Sample E	Sample F
	Median (IQR)	Median (IQR)	Median (IQR)	Median (IQR)	Median (IQR)	Median (IQR)
Al	293.9 (179–421)	236.3 (171–319)	184 (129–255)	589.7 (398–1680)	243.9 (217–291)	169.9 (124–633)
As	4.1 (2.7–5.3)	4.1 (2.6–5.3)	3.7 (2.9–5.0)	6.2 (5.6–9.2)	2.0 (1.5–2.2)	2.3 (2.0–2.6)
Cd	2.5 (1.7–4.0)	2.7 (1.6–3.7)	2.6 (1.6–3.5)	1.0 (0.7–2.1)	1.2 (0.7–1.9)	1.0 (0.5–1.2)
Co	31 (27–37)	8.2 (5.5–12)	10.9 (6.1–12)	13.2 (11–27)	16.6 (14–27)	4.4 (4.0–4.9)
Cr	337.8 (247–400)	268.6 (219–384)	307.7 (230–336)	174 (157–226)	341.8 (313–358)	278.5 (266–334)
Fe	350.4 (301–500)	315.4 (242–382)	378.1 (310–533)	903.2 (702–1225)	194.5 (112–373)	267 (245–376)
Mn	17.7 (15–22)	15.1 (13–19)	8.1 (6.8–12)	28.9 (22–43)	17.8 (13–19)	< LOD
Ni	49 (34–55)	51.2 (30–240)	46 (33–62)	92.8 (34–135)	35.8 (27–627)	42.4 (31–63)
Pb	37 (29–40)	16.5 (12–86)	< LOD	62.7 (49–66)	< LOD	< LOD
Sn	35.6 (28–56)	147.4 (135–167)	33.7 (21–110)	52.2 (30–65)	83.2 (34–419)	32.5 (18–109)
V	2.0 (1.8–2.8)	2.3 (1.7–3.1)	3.2 (2.1–4.1)	5.2 (4.2–6.2)	1.5 (1.2–2.2)	1.4 (1.0–1.7)
Zn	651.8 (517–901)	280.2 (202–609)	160.2 (141–258)	1666 (1158–2177)	322.4 (222–712)	1063 (927–1144)

< LOD – below limit of detection.

Mass fractions of Co were below 50 µg kg^− 1^, with low variation within individual sample sets but notable differences between product sets (Table 2). The measured Co values were significantly lower than those reported in our previous study^6^, though they overlapped with the mass fractions reported by Kubachka et al.^18^. In contrast, Cr mass fractions were consistent both within and between sample sets, averaging around 300 µg kg^− 1^. Compared to previous studies^6^, Cr demonstrated a narrow-measured range of the mass fractions in five of the six studied products. The highest mass fractions of both analytes were measured in Sample E, with Co at 91.5 ± 11 µg kg^− 1^ and Cr at 359.0 ± 16.2 µg kg^− 1^. The analysis further identified Al, Fe, Ni, Sn, and Zn in most of the studied samples. Both Al and Fe were most abundant in Sample D, reaching 2055 ± 359.4 µg kg^− 1^ and 1831 ± 1366 µg kg^− 1^, respectively. Nickel and Sn mass fractions were highest in Sample E, reaching 1140 ± 720.3 and 474.9 ± 48.1 µg kg^− 1^, respectively, while Zn was highest in Sample C at 6041 ± 11,847 µg kg^− 1^ (Table S1). A previous study on metal contamination in nicotine vape liquids reported Al mass fractions in vape cartridges ranging from 17.5 to 128 µg kg^− 1^^10^, which is substantially lower than the values observed in this study (range: 221–2055 µg kg^− 1^, Table S1). The mass fractions of Sn and Zn varied significantly across studies^6,10,18,20^; however, the values reported in Table 2 are within the previously published range. Nickel is commonly associated with filaments or resistance wires in vaping cartridges^13–15^ and the reported mass fractions in the literature vary significantly, 0.04 ± 0.016–677 ± 2.0 mg kg^− 1^^6^ or 0.05–477 ± 21 mg kg^− 1^^18^.

Manganese was consistently detected only in Sample D, with the highest detected value at 63.6 ± 55.8 µg kg^− 1^ (Table S1). In the remaining samples, Mn mass fractions were either below the LOD for the entire product set or detected sporadically. Vanadium was detected in four of the six product sets, with mass fractions typically below 5 µg kg^− 1^, which is consistent with previously published results^6,21^.

Five of the studied samples were housed in 510-thread cartridges, and of these, three (Samples A, B, and E) were marketed to contain ceramic heating coils/elements, while Sample D was in a closed-pod system cartridge. Ceramic coils are made of a porous ceramic material that either encases a resistance wire or functions as a heating element itself. It could be argued that ceramic coil designs may reduce the risk of metal contamination by minimizing direct contact between metallic components and the vaping liquid. However, the results obtained in this study (Table 2) do not support this argument, no significant differences in total mass fractions of metal typically found in coils (Al, Co, Cr, Ni, Zn) were observed between products utilizing 510-thread cartridges, regardless of the marketed coil material. Interestingly, the highest mass fractions of Sn were measured in Samples B and E, the samples that were marketed as being equipped with a ceramic heating element. In contrast, the closed-pod system used in Sample D employs a design in which the resistance wire remains fully submerged in the vape liquid. If the vape liquid facilitates corrosion of the metal components, then this design would be expected to exhibit higher levels of metal contamination. Indeed, Sample D contained the highest median for eight of the twelve measured metals, with Al, Fe, and Zn being the most abundant (Table 2). Another factor that may have impacted the concentration of metal contamination in Sample D could be the vaping liquid volume; it contained less than half the liquid of the other products and if leaching or corrosion rates between products is comparable, it would be expected that Sample D would have a correspondingly higher concentration of metal contamination in its smaller volume.

Although the Class 1 metals As, Cd, Hg, and Pb are the primary metals typically monitored for in vaping products, studies indicate that exposure to other metals through inhalation may also present health risks^22,23^. Using the U.S. Pharmacopoeia’s permitted levels of elemental impurities in inhaled products with a 10 g maximum daily dose (Table S2) as a reference^24^, Cr was found to exceed the concentration limit of 0.3 µg g^− 1^ in over 50% of the studied products, with at least one cartridge per lot exceeding this limit. In addition, Ni mass fractions exceeded the concentration limit of 0.5 µg g^− 1^ in two cartridges from different products (Table S1).

**Fig. 1:**
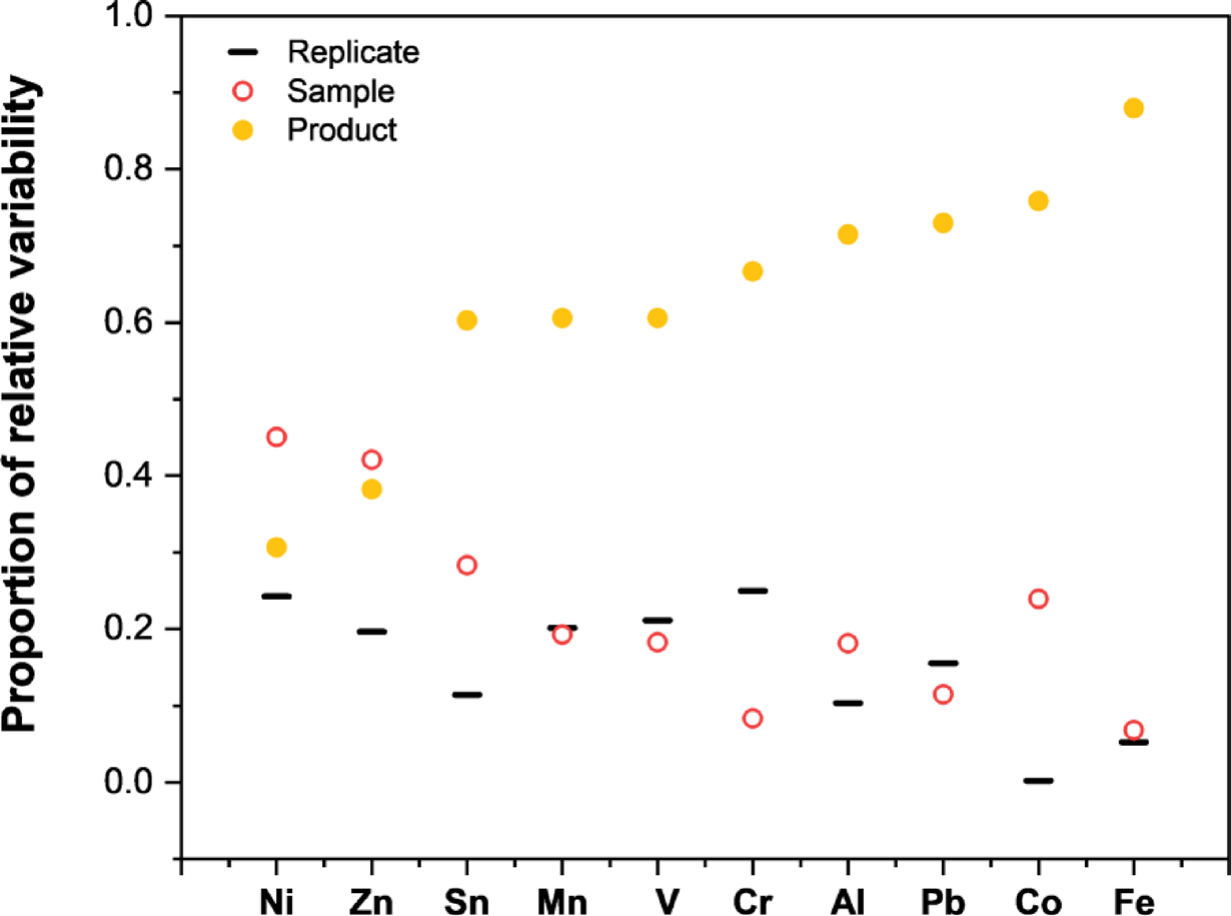
Comparison of relative variability across replicate samplings the same cartridge (solid line), variability between cartridges of the same production lot (open circle), and differences between distinct products (closed circle).

To assess variability between identical products, five vape cartridges from the same production lot were analyzed in triplicate. Additionally, to assess variation between different products, six different cannabis vape liquids were studied. Figure 1 illustrates comparisons of mass fractions among individual sampling replicates from each cartridge (solid line), between cartridges from the same production lot (open circles), and between different products (closed circles), as calculated by two-way ANOVA with replicates. When the solid line and open circles are closely aligned, as observed for Mn, V, Pb, and Fe, minimal within-batch variation was detected. However, most analytes exhibited some degree of variability, either within replicate samplings of the same cartridge (Cr) or between cartridges from the same production lot (Ni, Zn, Sn, Al, and Co). Furthermore, substantial variation was observed between different products for most elements, except for Ni and Zn, where within-batch variability was the primary source of variation. Elements that were detected close to the method LOD (As and Cd) were excluded from this comparison as their variability may primarily be driven by measurement uncertainty. The presented data suggests that the mass fractions of the studied elements are product-dependent, thus challenging general assumptions about metal content across cannabis vape liquids. This variability therefore poses concerns with regards to representative sampling protocols for regulatory testing, as compliance with Pharmacopeia-established maximum tolerated limits may not always be reliably ensured across all individual cartridges of a product within the same lot. Even if several cartridges from a single lot meet the established limits, the high degree of variation suggests there is a risk that others in the batch may not comply.

### Analysis of metal NPs in the cannabis vape liquids

The removal of Samples D and E from the vaping device presented challenges due to the device design. As a result, Sample D was extracted from three cartridges, while Sample E was obtained from two cartridges. The contents of each cartridge in these cases were analyzed individually, and as shown in Table 3, all studied samples contained varying numbers of metal particles. For a sample to be quantified in terms of particle number and size, the instrument needed to detect > 100 particles per acquisition time, which is the minimum number of particles required to be detected for optimal counting conditions in ICP-MS^25^. Therefore, samples in which the detected particle count was below this minimum number of detectable particles (MNDP) could not be quantified. However, this does not imply that these samples were free of metal particles.

**Table 3:** Median size (nm), limits of detection (LOD, nm), and calculated number of detected NPs in the studied cannabis vape devices.

Analyte	Sample ID	Median size (nm)	LOD (nm)	NPs/device	Analyte	Sample ID	Median size (nm)	LOD (nm)	NPs/device
Fe	A	71	34	2.96 × 10^6^	Co	A	NQ	NQ	< MNDP
	B	54	38	6.99 × 10^5^		B	NQ	NQ	< MNDP
	C	69	32	4.38 × 10^7^		C	NQ	NQ	< MNDP
	D_1	47	22	2.57 × 10^7^		D_1	NQ	NQ	< MNDP
	D_2	49	22	9.14 × 10^5^		D_2	NQ	NQ	< MNDP
	D_3	45	21	5.82 × 10^6^		D_3	NQ	NQ	< MNDP
	E_1	46	22	4.78 × 10^6^		E_1	NQ	NQ	< MNDP
	E_2	48	22	2.57 × 10^7^		E_2	NQ	NQ	< MNDP
	F	51	25	5.72 × 10^6^		F	38	20	1.28 × 10^5^
Ni	A	65	32	3.56 × 10^5^	Zn	A	79	42	2.97 × 10^5^
	B	NQ	NQ	< MNDP		B	NQ	NQ	< MNDP
	C	63	32	1.39 × 10^6^		C	69	38	5.05 × 10^5^
	D_1	69	32	7.28 × 10^5^		D_1	75	38	1.20 × 10^6^
	D_2	NQ	NQ	< MNDP		D_2	NQ	NQ	< MNDP
	D_3	NQ	NQ	< MNDP		D_3	NQ	NQ	< MNDP
	E_1	NQ	NQ	< MNDP		E_1	NQ	NQ	< MNDP
	E_2	69	32	2.20 × 10^6^		E_2	91	43	3.74 × 10^6^
	F	62	31	5.40 × 10^5^		F	85	44	4.61 × 10^5^
Al	A	63	35	3.80 × 10^6^	Cr	A	NQ	NQ	< MNDP
	B	61	42	3.19 × 10^6^		B	NQ	NQ	< MNDP
	C	66	35	3.18 × 10^6^		C	49	23	2.69 × 10^7^
	D_1	65	35	6.25 × 10^6^		D_1	NQ	NQ	< MNDP
	D_2	64	34	9.51 × 10^5^		D_2	NQ	NQ	< MNDP
	D_3	63	34	2.22 × 10^6^		D_3	NQ	NQ	< MNDP
	E_1	62	34	2.26 × 10^6^		E_1	NQ	NQ	< MNDP
	E_2	66	35	2.43 × 10^7^		E_2	NQ	NQ	< MNDP
	F	61	35	3.54 × 10^6^		F	NQ	NQ	< MNDP
Cu	A	60	27	1.47 × 10^6^	Sn	A	61	28	2.86 × 10^5^
	B	NQ	NQ	< MNDP		B	NQ	NQ	< MNDP
	C	56	25	1.22 × 10^6^		C	NQ	NQ	< MNDP
	D_1	52	25	4.60 × 10^6^		D_1	NQ	NQ	< MNDP
	D_2	55	25	9.64 × 10^5^		D_2	NQ	NQ	< MNDP
	D_3	53	25	9.40 × 10^5^		D_3	NQ	NQ	< MNDP
	E_1	61	27	7.84 × 10^5^		E_1	NQ	NQ	< MNDP
	E_2	54	26	1.05 × 10^7^		E_2	64	28	1.41 × 10^6^
	F	60	27	2.38 × 10^6^		F	NQ	NQ	< MNDP

NQ – not quantified due to < 100 particles detected per acquisition time; < MNDP – below minimum number of detected particles.

Most of the studied analytes were detected in all samples, although not all were quantifiable, as the number of detected particles did not always exceed the MNDP. No particles containing V and Pb were detected in any of the studied samples. The median size for all detected particles was below 100 nm, although the collected data also indicated the presence of particles > 100 nm. The particle number concentration (PNC) was similar to previously reported concentrations^7^; however, the range of PNC was narrower in the present study. When comparing products fitted with different coil designs, the PNC for some analytes (Al, Cu, Co, Sn, and Zn) was highest in one of the replicates of Sample E, which was marketed as being equipped with a ceramic heating element. Conversely, the PNC in samples from the closed-pod system (Sample D) was not significantly different from that in 510-thread designs (Table 2).

Analysis of multiple identical samples (3 × Sample D and 2 × Sample E) demonstrated that, although the median size of detected metal particles remained fairly consistent (average SD ± 3 nm), the PNC varied by orders of magnitude. For example, in Sample D, the PNC for Fe ranged from 9.14 × 10^5^ to 2.57 × 10^7^, while in Sample E, the PNC for Cu varied between 7.84 × 10^5^ and 1.05 × 10^7^. This variation in PNC directly impacts the total metal mass fraction measured in the digested samples and contributes to the within-batch variation, as previously suggested^6^. Furthermore, these observations raise questions regarding the appropriate sample size for representative sampling of consumer products of this type, which may warrant further investigation.

It should be noted that the exact composition of the measured metal particles is not known, as the ICP-MS instrument used in this study is not capable of simultaneous detection of multiple elements per scan. Additionally, particle size calculations are based on the assumption of a spherical diameter and a known density, which presumes that the particles are made of single elements. As a result, the reported particle sizes should be treated as information values only and may be underestimates of actual particle sizes in the event particles contain more than one element.

### Cannabis aerosol particles analysis

**Fig. 2:**
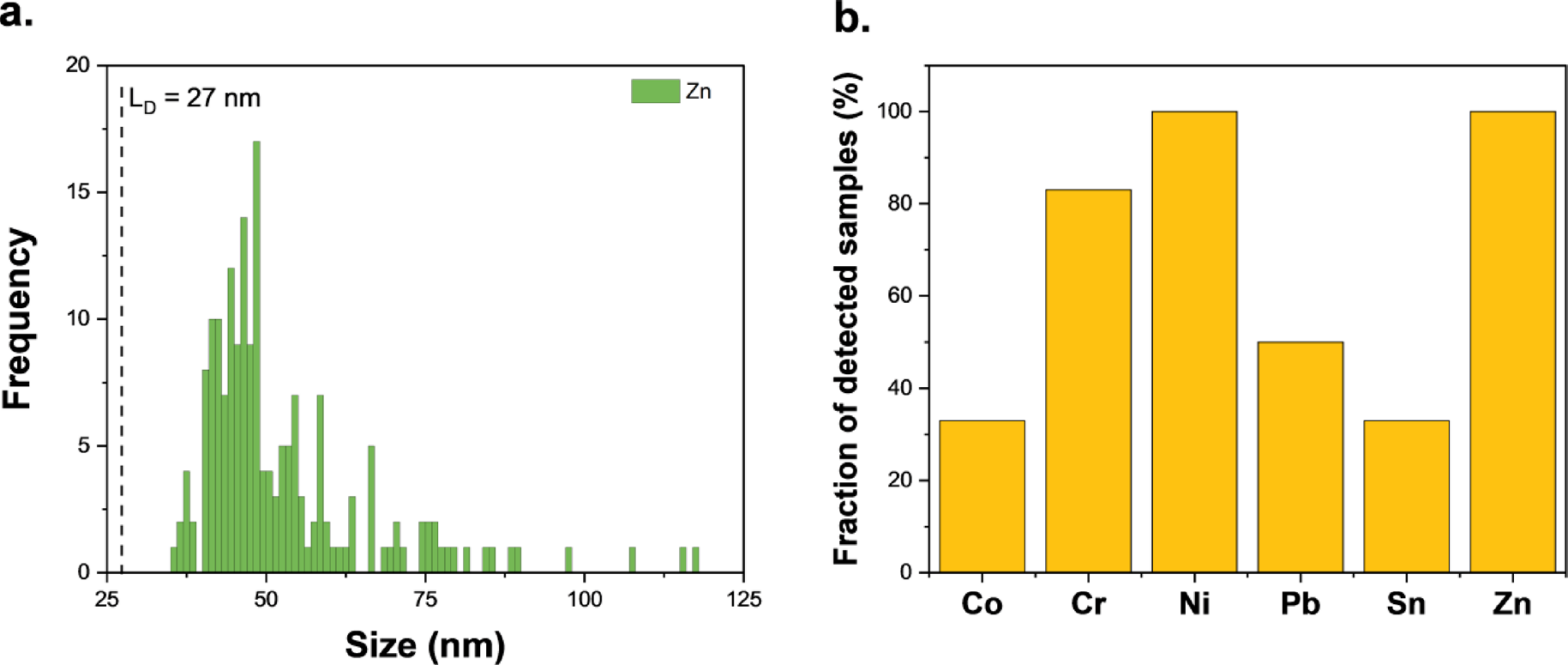
Off-line analysis of cannabis aerosol by sp-ICP-MS. (**a**) Particle size distribution of Zn-containing particles in the aerosol generated from Sample C and (**b**) the fraction of aerosol samples in which particles containing specific analytes were detected.

The analysis of metal particles in cannabis vape liquids revealed significant variation in PNC both within and between product batches, making any methodological approach to aerosol analysis extremely challenging. There is no guarantee that two identical products will contain similar PNC values, and no direct correlation can be established between the PNC in cannabis vape liquid removed from a cartridge and that in the generated aerosol, as measurements cannot be performed on the same device. Therefore, the aim of the aerosol analysis was to determine whether metal particles present in cannabis vape liquids can be transported into the aerosol generated during vaping. The vaping session for 510-thread cartridges consisted of 150 puffs, resulting in an average consumption of 0.35 g of cannabis vape liquid. The closed-pod system cartridges were designed to operate at lower temperature settings, requiring vaping sessions with a higher number of puffs to aerosolize a higher mass of cannabis liquid. However, even with 250 puffs, the average mass of consumed cannabis liquid was only 0.063 g. As shown in Fig. 2a, metal particles were detected in the collected aerosol; however, the PNC in all samples was below the MNDP. Additionally, Fe, Al, and Cu particles measured in the aerosols could not be distinguished from the particles present in the blank samples and were therefore excluded from the discussion. Aerosol generated from all samples contained Ni and Zn particles, while Cr particles were detected in 80% of the studied samples. Particles containing Co, Pb, and Sn were detected in ≤ 50% of vaped cartridges (Fig. 2b).

The generated aerosol was collected in two open-ended impingers connected in tandem, containing an organic solvent (PremiSolv) miscible with cannabis vaping liquids. During preliminary experiments, the contents of the second impinger were analyzed for metal particles; however, none were detected. Consequently, only the contents of the first impinger were reported. Previously published studies have used two techniques for collecting cannabis aerosol: tandem impingers or a condensation tube^19,26^. In both studies, acetone was used either as the collection solvent in the impingers or as a washing solvent for the condensation tube. However, the authors reported clogging of the fritted impinger with cannabis matrix, which could be expected as the cannabis matrix is not miscible with such a polar solvent. Similarly, washing the condensation tube with acetone may not have effectively removed all of the condensed aerosol from the tube walls, potentially leading to an underestimation of metal content. Additionally, the high vapor pressure of acetone (31 kPa) required keeping the impingers in an ice bath, which subsequently cooled the glassware and may have led to condensation points along the aerosol pathway. In contrast, the solvent used in the current study had a significantly lower vapor pressure (0.03–0.06 kPa), allowing aerosol collection to be conducted at room temperature. Furthermore, a preliminary visual examination of the connectors and tubing pathways leading to the impinger showed no condensation points or aerosol accumulation.

In previous studies, cannabis aerosol was subjected to acid digestion followed by total metal quantitation, without analysis of metal particles^19,26^. The only published study analyzing metal particles generated by vaping devices was performed on nicotine products^16^. The authors reported significantly higher PNCs detected in aerosols than those observed in the current work. However, as discussed earlier, direct comparison of PNC values between published research is not feasible due to the large within- and between-batch and product variations demonstrated in this study, in addition to the matrix induced differences between cannabis and nicotine vape liquids.

Users of cannabis vape liquids may be exposed to metals in two physical forms: ionic and particulate. Ionic metals may leach from the hardware components or originate as contaminants introduced during cannabis vape liquid production, storage, or filling of the cartridge. These metal ions can evaporate directly^27^ and may subsequently enter the aerosol phase via recondensation as vapor cools upon exiting the vape chamber^28^. In contrast, particulate metals, being non-volatile, are unlikely to undergo evaporation-recondensation. Instead, these particulate metals likely enter the aerosol phase through bubble bursting.

Bubble bursting is when, during vaping, bubbles form within milliseconds at the heated surface of the vape liquid, where local temperatures rise only a few kelvin above the liquid’s boiling point^29^, via nucleate boiling. Nucleate boiling can occur even at relatively low power settings, such as the 5–10 W used in cannabis vape devices (power was calculated using Ohm’s law, P = V^2^/R, with typical values of *R* = 1.5 Ω and V = 2.8–3.8 V, for the battery). Rapid heating of the coil results in extremely heterogeneous temperature distributions^30^, promoting nucleate bubbling. These bubbles detach from the heater-liquid interface, travel to the air-liquid interface, and burst, generating airborne liquid droplets (aerosols)^31^. Some of these aerosols may contain particulate metals. This hypothesized bubble-bursting mechanism is supported by data from Floyd et al.^32^, who observed a nonlinear increase in the relative number of > 0.5 μm aerosol particles with increasing vaping power, while the size of approximately 0.2 μm aerosol particles remained constant. This discrepancy implies a shift in the physical mechanism of aerosol particle generation, such as increased bubble generation and bursting, as opposed to a simple linear increase in condensation.

Previous work on nicotine vape devices has shown that metal emissions from the heating element increases as the device’s power supply increases^30^. This finding aligns with the larger aerosols observed by Floyd et al.^32^ and further supports the proposed bubble-bursting mechanism. Therefore, it can be inferred that operating cannabis vape devices at lower power settings likely reduces exposure to inhaled particulate metals.

### Scanning electron microscopy (SEM) of unused cartridge components

**Fig. 3:**
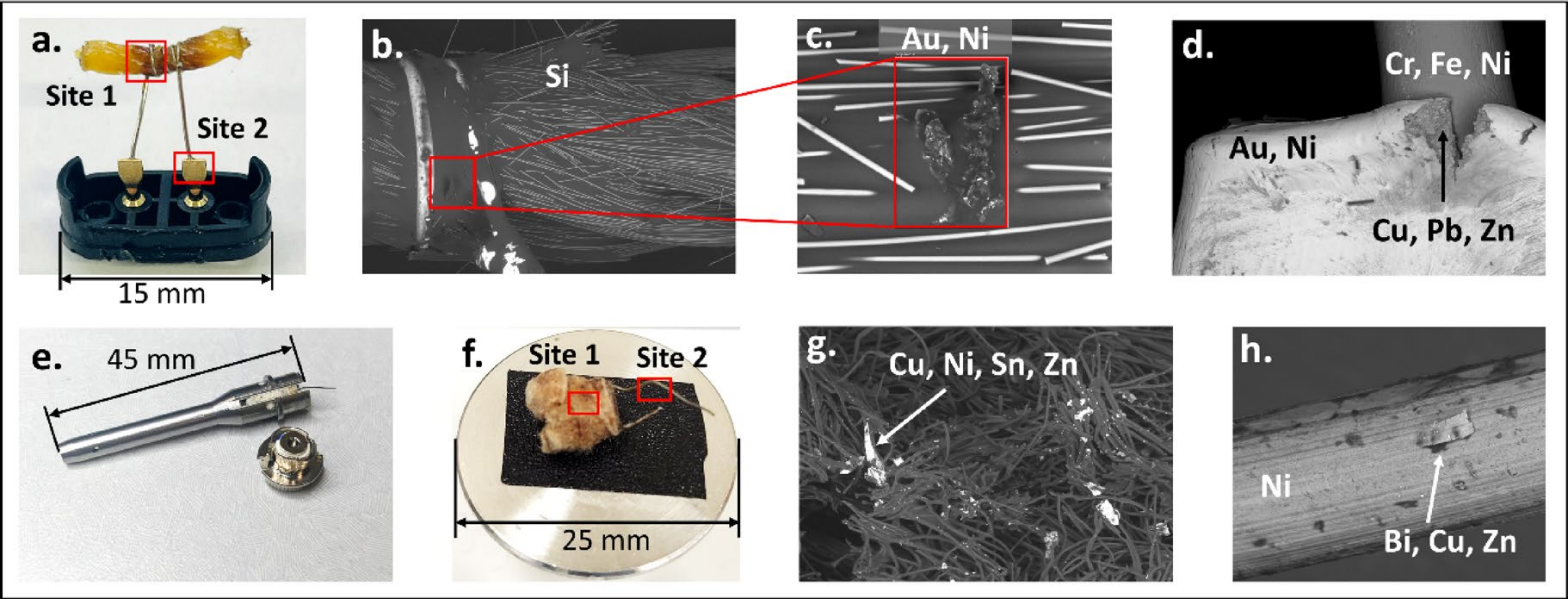
Elemental analysis of Sample D (closed-pod system) and Sample F (510-thread system) by SEM-EDS. (**a**) Metal components of the closed-pod system mounted on the SEM holder, with Site 1 (contact between the wick and heating wire) and Site 2 (connector pin) indicated for analysis. (**b**) SEM backscattered electron (BSE) image of Site 1. (**c**) Close-up of detected metal particles within the wick. (**d**) SEM image of the connector pin showing a surface crack. (**e**) Inner channel of the 510-thread cartridge, showing an incision for access to the wicking material. (**f**) Wicking material with the heating wire mounted on the SEM holder, with Site 1 and Site 2 indicated for analysis. (**g**) SEM image of Site 1, showing wicking material with incorporated metal particles. (**h**) SEM image of the heating wire, displaying a metal flake on the surface.

Individual vape cartridges were disassembled for component analysis using SEM, with subsequent elemental analysis via energy dispersive X-ray spectroscopy (EDS). According to the manufacturer specifications, Samples A, B and E were expected to contain ceramic heating coils; however, their components did not appear different from those of the other disassembled 510 thread cartridges. Samples D (closed-pod system) and F (510-thread cartridge) were analyzed to identify possible metal particles and determine the elemental composition of individual cartridge components. As shown in Fig. 3a, two key sites on Sample D were closely examined. Site 1, the contact interface between the wick and heating wire, and Site 2, the connector pin linking the battery and the heating wire. The wick was heavily coated with cannabis vape liquid, which made finding metal particles within the matrix challenging. The matrix was intentionally left intact to avoid removing any particles that may have been lodged in the wick structure. Despite the matrix coating, a particle containing Au and Ni was identified on the surface of the wick (Fig. 3b,c). The metal composition of this particle corresponded with that of the connector pins, which were plated with Au and Ni. Closer examination of the connector pins revealed cracking on the surface (Fig. 3d). Elemental analysis within the crack, representing the core material of the connector pins, identified an alloy composed of Cu, Pb, and Zn. The heating wire itself has shown inconsistencies in its elemental composition. The parts that were wrapped around the wick were composed of Fe, Cr, and Ni, however further away from the wick, closer to the connector pins, the analysis have shown primarily Ni (Figure S1 and S2). Thus, it is possible that the heating wire was Ni plated, and the coating was heterogeneous or the Ni coating was partially corroded in the area wrapped around the wick, what resulted in leaching of metals into the cannabis vape liquid. The metal composition of these cartridge parts correlated well with the metal particles found in the aerosol of Sample D, mainly Zn, Ni, and Pb. As it can be seen in Table 3, Cu particles were also present in the cannabis vape liquids; however, they could not be distinguished from the blank in the collected aerosol.

Disassembly of Sample F, a 510-thread cartridge, was more challenging due to the design of the central channel, which required cutting (Fig. 3e) to gain access to the heating wire and wick. The wicking material, along with the heating wire, was mounted on the SEM holder, and two sites were examined (Fig. 3f). Site 1 corresponded to the wicking material, which was generally free of the hydrocarbon cannabis vaping liquid matrix, while Site 2 corresponded to the heating wire. As shown in Fig. 3g, the wicking material contained numerous metal particles, visible as brightly illuminated spots in the SEM image. The main elemental composition of these particles consisted of Cu, Ni, Sn, and Zn, while the wicking material itself was most probably made of cotton fibers. The surface of the heating wire was composed of Ni (Fig. 3h); however, small flake-like particles containing Cu, Zn, and Bi were also observed on its surface.

The composition of the metal components was consistent with previous reports on both cannabis^13^ and nicotine vape devices^13,15,16,33^. The metal particles detected in the studied cannabis vape liquids by sp-ICP-MS consisted of the same elements as those found in the vaping cartridges, strongly suggesting that contamination originated from the device itself. However, the exact mechanism of contamination remains unclear. It is possible that the metal particles observed in the SEM images of Sample F (Fig. 3g) resulted from contamination introduced during the disassembly process, particularly due to the incision into the inner channel of the cartridge to access the wicking material. However, in Sample D, which did not require forceful manipulation, metal particles were still found on the wick surface. A crack was observed on the connector pin of Sample D, and possible signs of corrosion were found. Cracking on the connector of nicotine vape devices has been previously reported, along with the detection of large numbers of metal particles in collected aerosols, with corrosion becoming evident after device use^16^. Other studies have also shown increased metal concentrations in nicotine vape liquids following a vaping session^14,19^. These findings suggest that device operation can influence the stability of metal components. However, this would not account for the presence of metal particles in unused cartridges containing cannabis vape liquids. It is possible that some mechanical abrasion may occur during the manufacturing process, such as during alloy machining/casting, assembly of the hardware, and/or cutting of the resistance wire. Such contamination introduced during cartridge manufacturing is likely not removed prior to filling. These factors may lead to metal contamination of the vape liquids prior to vaping, requiring further investigation.

## Conclusion

Cannabis vape liquids are increasingly popular cannabis products^34^, but their consumption may have adverse health effects yet to be characterized. These liquids contain metal particles of varying sizes and compositions, which are transported to consumers in the generated aerosol and inhaled. Given the metal composition of these particles, it is highly likely that they originate from the metal components of the vape devices. Furthermore, surface cracking observed in the studied products suggests that further metal leaching may also occur during device use. The presence of metal particles complicates comparisons of total metal mass fractions both between and within production batches, as these levels are not determined by the manufacturing of the cannabis vape liquids themselves but rather by the quality of the vaping hardware. Findings to date show that the quality of nicotine vaping devices has not improved over the time, highlighting the importance of continued quality control and monitoring of cannabis vaping devices. Results presented in this study have shown contamination of cannabis vape liquids with metal particles containing Al, Co, Cr, Cu, Ni, Sn, and Zn. Particles containing some of these metals were also found in the collected aerosols, highlighting the importance of characterizing these metal hazards in cannabis vaping liquids to ensure the risks posed by them can be properly understood and addressed where necessary. Importantly, the concern is not merely the presence of metal particles in the liquid but their transport in the aerosol and subsequent inhalation, which may pose health risks that remain to be fully characterized and understood. Development and selection of devices that minimize or eliminate metal transport into the aerosol and ultimately to consumers should be considered. Additionally, ensuring accurate product marketing, such as heating coil composition, is important as this information may be a pre-determining factor for consumer choice and product safety. The mismarketing of coil types observed in this study not only limited our ability to compare different product designs but also raised broader concerns about the reliability and transparency of product marketing within the industry. Addressing these inconsistencies is essential to improving consumer safety, ensuring product performance, and strengthening regulatory compliance.

## Supplementary Information

Below is the link to the electronic supplementary material.

Supplementary Material 1

## Data Availability

All data generated or analyzed during this study are included in this published article (and its Supplementary Information files).
