# Supplementary material for: Tracking metal presence in cannabis vaping products from source to inhalation
Source: Sci Rep. 2025 Aug 29;15:31939. doi: 10.1038/s41598-025-17004-2 (PMC12397267; doi:10.1038/s41598-025-17004-2)
Supplement: Supplementary file 1 — Supplementary Material 1 [file 41598_2025_17004_MOESM1_ESM.docx]

**Tracking metal presence in cannabis vaping products from source to inhalation**

Zuzana Gajdosechova^1^*, Joshua Marleau-Gillette^1^, Matthew Polivchuk^2^, Ivana Kosarac^3^, Guru Prasad Katuri^3^, Dharani Das^3^, Ashley Cabecinha^4^, Andrew Waye^4^, Hanan Abramovici^4^

^1^ Metrology Research Center, National Research Council Canada, 1200 Montreal Rd., Ottawa, ON, K1A 0R6, Canada

^2^ Natural Resource Canada, 601 Booth St., Ottawa, ON, K1A 0E8, Canada

^3^ Tobacco Control Directorate, Controlled Substances and Cannabis Branch, Health Canada, 150 Tunney’s Pasture Driveway, Ottawa, ON K1A 0K9, Canada

^4^ Office of Cannabis Science and Surveillance, Controlled Substances and Cannabis Branch, Health Canada, 150 Tunney’s Pasture Driveway, Ottawa, ON, K1A 0K9, Canada

* Corresponding author: [Zuzana.Gajdosechova@nrc-cnrc.gc.ca](mailto:Zuzana.Gajdosechova@nrc-cnrc.gc.ca)

**Supporting Information**

**Table S1.** Mass fractions (µg kg^-1^) of metals in cannabis vape liquids (n ≥ 3).

| **Product ID** |  | **Al** | **As** | **Cd** | **Co** | **Cr** |
| --- | --- | --- | --- | --- | --- | --- |
| **Sample A** | A_1 | 253.3 ± 62.7 | 3.8 ± 1.9 | 2.6 ± 2.1 | 29.9 ± 6.9 | 327.5 ± 86.6 |
|  | A_2 | 177.9 ± 89 | 4.0 ± 1.4 | 2.5 ± 1.3 | 32.1 ± 3.8 | 322.5 ± 92.9 |
|  | A_3 | 484.3 ± 49.4 | 4.2 ± 1.7 | 2.7 ± 1.8 | 29.9 ± 3.2 | 319.3 ± 63.7 |
|  | A_4 | 513.4 ± 413.8 | 4.6 ± 1.1 | 2.5 ± 1.2 | 43.0 ± 4.3 | 329.3 ± 113.6 |
|  | A_5 | 221.0 ± 75.7 | 3.5 ± 1.5 | 2.2 ± 1.1 | 22.9 ± 3.6 | 331.4 ± 122.1 |
| **Sample B** | B_1 | 226.4 ± 63.1 | 4.7 ± 1.8 | 2.2 ± 1.1 | 8.8 ± 3.9 | 306.1 ± 90.8 |
|  | B_2 | 165.2 ± 55.4 | 4.9 ± 1.5 | 3.1 ± 2.6 | 8.7 ± 3.6 | 303.0 ± 81.6 |
|  | B_3 | 271.0 ± 76.3 | 3.8 ± 1.6 | 2.2 ± 1.3 | 8.0 ± 3.4 | 304.7 ± 103 |
|  | B_4 | 924.6 ± 1356.5 | 3.8 ± 1.4 | 2.4 ± 1.2 | 9.3 ± 3.9 | 305.9 ± 100.1 |
|  | B_5 | 379.4 ± 251.8 | 3.7 ± 1.5 | 2.2 ± 1.2 | 8.6 ± 3.1 | 258.8 ± 95.1 |
| **Sample C** | C_1 | 418.6 ± 382.7 | 3.9 ± 1.3 | 2.4 ± 1.0 | 8.6 ± 3.5 | 262.8 ± 57.5 |
|  | C_2 | 150.5 ± 70.6 | 4.2 ± 1.1 | 2.2 ± 1.0 | 8.9 ± 3.2 | 301.5 ± 80.4 |
|  | C_3 | LOD | 4.3 ± 1.3 | 2.2 ± 1.2 | 8.8 ± 2.9 | 300.0 ± 70.9 |
|  | C_4 | 180.5 ± 64.4 | 3.8 ± 1.1 | 2.0 ± 0.9 | 12.0 ± 6.5 | 312.1 ± 17.6 |
|  | C_5 | 222.1 ± 138 | 3.6 ± 0.9 | 2.2 ± 0.8 | 16.9 ± 14.7 | 286.2 ± 75.0 |
| **Sample D** | D_1 | 842.2 ± 726.2 | 8.3 ± 3.5 | 3.2 ± 0.0 | 18.5 ± 8.6 | 206.0 ± 64.0 |
|  | D_2 | 641.2 ± 445.8 | 9.2 ± 3.4 | LOD | 15.0 ± 8.0 | 185.8 ± 23.7 |
|  | D_3 | 2055 ± 359.4 | 5.5 ± 1.1 | LOD | 11.1 ± 2.6 | 237.3 ± 186.9 |
|  | D_4 | 425.9 ± 152 | 5.2 ± 1.0 | LOD | 10.7 ± 0.4 | 172.8 ± 47.7 |
|  | D_5 | 1092.6 ± 763.8 | 17.6 ± 20.4 | LOD | 24.4 ± 22.2 | 324.1 ± 253.3 |
| **Sample E** | E_1 | 399.5 ± 237.9 | 2.1 ± 0.7 | LOD | 91.5 ± 11 | 359.0 ± 16.2 |
|  | E_2 | 137.0 ± 85.9 | 2.1 ± 0.6 | LOD | 16.3 ± 0.4 | 344.1 ± 12.9 |
|  | E_3 | 248.7 ± 43.5 | 1.8 ± 0.2 | LOD | 26.5 ± 1.6 | 308.5 ± 60.5 |
|  | E_4 | 120.9 ± 46.1 | 1.5 ± 0.7 | 1.9 ± 0 | 10.7 ± 0.6 | 339.8 ± 45.1 |
|  | E_5 | LOD | 1.9 ± 0.4 | LOD | 14.6 ± 0.9 | 301.1 ± 74.4 |
| **Sample F** | F_1 | LOD | 1.9 ± 1 | LOD | 4.1 ± 0.3 | 276.1 ± 101.5 |
|  | F_2 | 568.7 ± 692.1 | 2.5 ± 0.8 | LOD | 4.1 ± 0.2 | 271.3 ± 6.4 |
|  | F_3 | 105.7 ± 88.7 | 2.1 ± 0.6 | LOD | 4.4 ± 0.4 | 284.4 ± 23.7 |
|  | F_4 | LOD | 2.3 ± 0.2 | LOD | 7.2 ± 4.7 | 305 ± 75.3 |
|  | F_5 | LOD | 2.4 ± 0.2 | LOD | 5.2 ± 0.5 | 313.4 ± 81.5 |
| **Method LOD** |  | 105 | 0.82 | 0.49 | 2.9 | 16.8 |

**Table S1.** continuation

| **Product ID** |  | **Fe** | **Mn** | **Ni** | **Pb** | **Sn** |
| --- | --- | --- | --- | --- | --- | --- |
| **Sample A** | A_1 | 290.8 ± 97.8 | 14.2 ± 6.7 | 52.3 ± 3.9 | 30.7 ± 8.7 | 52.9 ± 39.8 |
|  | A_2 | 370 ± 138.4 | 14.6 ± 8.7 | 56.0 ± 34.3 | LOD | 35.6 ± 16.5 |
|  | A_3 | 420.5 ± 123.5 | 14.0 ± 5.1 | 37.8 ± 11.5 | LOD | 55.5 ± 25.1 |
|  | A_4 | 430.6 ± 105.9 | LOD | 57.0 ± 9.2 | 38.2 ± 1.5 | 38.7 ± 8.2 |
|  | A_5 | 489.1 ± 383.6 | LOD | 25.3 ± 7.4 | LOD | 30.5 ± 8.3 |
| **Sample B** | B_1 | 287.8 ± 69.1 | LOD | 29.1 ± 11.6 | 66.9 ± 53.5 | 173.8 ± 56.2 |
|  | B_2 | 277.2 ± 81.4 | LOD | 43.0 ± 29.5 | 49.8 ± 77.1 | 173.7 ± 62.2 |
|  | B_3 | 288.4 ± 147 | LOD | 160.8 ± 220.4 | LOD | 128.9 ± 8.5 |
|  | B_4 | 269.1 ± 57.6 | LOD | 102.7 ± 173.1 | 6.1 ± 7 | 174.6 ± 10.5 |
|  | B_5 | 444.7 ± 196.2 | 13.0 ± 8.5 | LOD | LOD | 148.0 ± 12.4 |
| **Sample C** | C_1 | 378.3 ± 152.1 | LOD | 28.2 ± 4.6 | 5.6 ± 8.6 | LOD |
|  | C_2 | 544.8 ± 149.5 | LOD | 55.8 ± 7.9 | LOD | 54.6 ± 57.6 |
|  | C_3 | 434.9 ± 189.4 | LOD | 57.1 ± 24.6 | LOD | LOD |
|  | C_4 | 322.2 ± 47.9 | LOD | 39.9 ± 15.2 | LOD | LOD |
|  | C_5 | 326 ± 121.4 | LOD | 601.8 ± 1143.9 | LOD | 69.5 ± 102.3 |
| **Sample D** | D_1 | 985.7 ± 151.5 | 63.6 ± 55.8 | 89.4 ± 21.6 | 69.3 ± 6.2 | 51.0 ± 26.0 |
|  | D_2 | 821.0 ± 375.4 | 29.0 ± 6.9 | 47.0 ± 43.0 | 48.9 ± 1.7 | 45.7 ± 18.1 |
|  | D_3 | 1128 ± 412.5 | 31.5 ± 11.6 | 64.7 ± 70.7 | 65.0 ± 1.6 | 201.8 ± 271.5 |
|  | D_4 | 748.1 ± 285.8 | 22.8 ± 4.3 | 113.2 ± 93.7 | 60.4 ± 4.5 | 42.2 ± 18.9 |
|  | D_5 | 1831 ± 1366 | 61 ± 43.7 | 121.2 ± 181 | 50.6 ± 11.2 | 73.3 ± 51.2 |
| **Sample E** | E_1 | 314.5 ± 134.6 | 14.2 ± 4.6 | 1140 ± 720.3 | LOD | 474.9 ± 48.1 |
|  | E_2 | 233.5 ± 124.6 | LOD | 24.4 ± 6.4 | LOD | 37.0 ± 2.8 |
|  | E_3 | 235.1 ± 194.9 | LOD | 29.4 ± 6.5 | LOD | 115.4 ± 3.5 |
|  | E_4 | LOD | LOD | LOD | LOD | 43.5 ± 34.4 |
|  | E_5 | 187.2 ± 188.7 | LOD | 31.9 ± 16.3 | LOD | 250 ± 373.5 |
| **Sample F** | F_1 | 254.5 ± 74.8 | LOD | LOD | LOD | 43.9 ± 56.0 |
|  | F_2 | 280.8 ± 106.6 | LOD | LOD | LOD | 106.3 ± 155.2 |
|  | F_3 | 357.9 ± 99.7 | LOD | 35.4 ± 25.1 | LOD | 24.3 ± 24.7 |
|  | F_4 | 266.9 ± 50.7 | LOD | 33.5 ± 13.5 | LOD | 16.9 ± 14.0 |
|  | F_5 | 288.2 ± 132.3 | LOD | 70.8 ± 78.5 | LOD | LOD |
| **Method LOD** |  | 175.8 | 12 | 23.3 | 5.5 | 15.6 |

**Table S1.** continuation

| **Product ID** |  | **V** | **Zn** |
| --- | --- | --- | --- |
| **Sample A** | A_1 | LOD | 739 ± 202 |
|  | A_2 | 2.2 ± 0.6 | 685 ± 53.8 |
|  | A_3 | 2.2 ± 0.5 | 531.1 ± 17.1 |
|  | A_4 | 2.4 ± 0.6 | 1073 ± 193 |
|  | A_5 | 2.5 ± 1.2 | 355.3 ± 59 |
| **Sample B** | B_1 | 2.4 ± 0.8 | 1091 ± 865 |
|  | B_2 | 2.5 ± 0.8 | 855 ± 1290 |
|  | B_3 | 2.6 ± 0.9 | 225.6 ± 84.2 |
|  | B_4 | 2.6 ± 0.7 | 334.3 ± 219.6 |
|  | B_5 | 2.3 ± 0.8 | 504.5 ± 450.8 |
| **Sample C** | C_1 | 2.9 ± 1.7 | LOD |
|  | C_2 | 4.3 ± 0.4 | 160.4 ± 110.4 |
|  | C_3 | 3.3 ± 1.8 | LOD |
|  | C_4 | 3.0 ± 1.0 | LOD |
|  | C_5 | 3.0 ± 0.8 | 6041 ± 11847 |
| **Sample D** | D_1 | 6.2 ± 2.7 | 3230 ± 1926 |
|  | D_2 | 5.3 ± 0.8 | 1831 ± 760 |
|  | D_3 | 5.0 ± 1.0 | 1581 ± 239 |
|  | D_4 | 4.3 ± 1.3 | 1242 ± 264 |
|  | D_5 | 12.1 ± 11.5 | 1429 ± 428 |
| **Sample E** | E_1 | 2.6 ± 0.4 | 985 ± 166 |
|  | E_2 | LOD | 337.9 ± 57.9 |
|  | E_3 | LOD | 215.8 ± 29.4 |
|  | E_4 | LOD | 214.7 ± 94.8 |
|  | E_5 | LOD | 439.2 ± 249.8 |
| **Sample F** | F_1 | LOD | 1074 ± 65.6 |
|  | F_2 | LOD | 942.9 ± 47.8 |
|  | F_3 | LOD | 1151 ± 384.7 |
|  | F_4 | LOD | 1129 ± 74.9 |
|  | F_5 | LOD | 1063 ± 147.6 |
| **Method LOD** |  | 2.2 | 129 |

**Table S2.** U.S. Pharmacopoeia’s permitted concentration of elemental impurities of metals relevant for the presented study. These concentration limits are intended for assessment of elemental impurities in drugs with daily dose of not more than 10 grams per day [1].

| **Element** | **Class** | **Inhalation concentration (mg/kg)** |
| --- | --- | --- |
| **Cd** | 1 | 0.3 |
| **Pb** | 1 | 0.5 |
| **As** | 1 | 0.2 |
| **Hg** | 1 | 0.1 |
| **Co** | 2A | 0.3 |
| **V** | 2A | 0.1 |
| **Ni** | 2A | 0.5 |
| **Pd** | 2A | 0.1 |
| **Cu** | 3 | 3 |
| **Cr** | 3 | 0.3 |


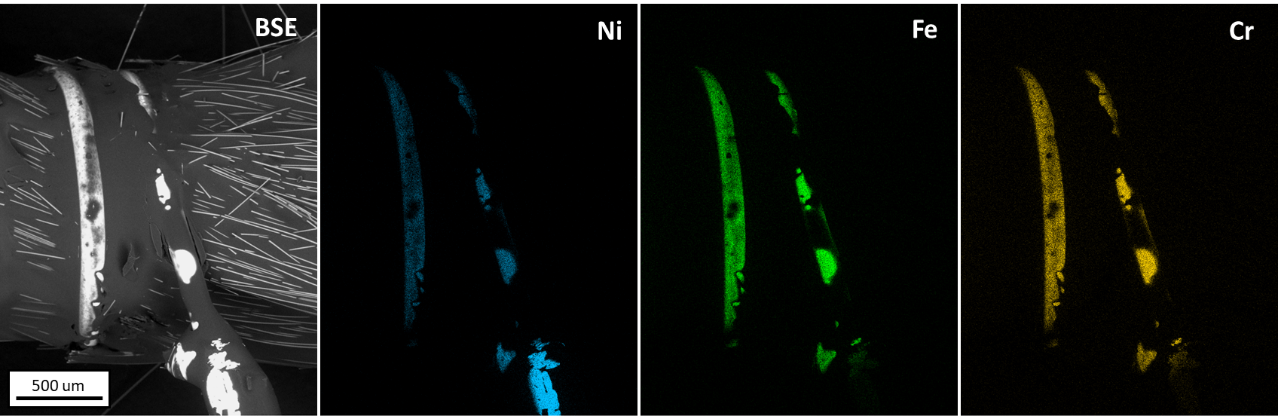


**Figure S1.** SEM-EDS backscatter electron (BSE) and chemical intensity maps of Sample D Site 1


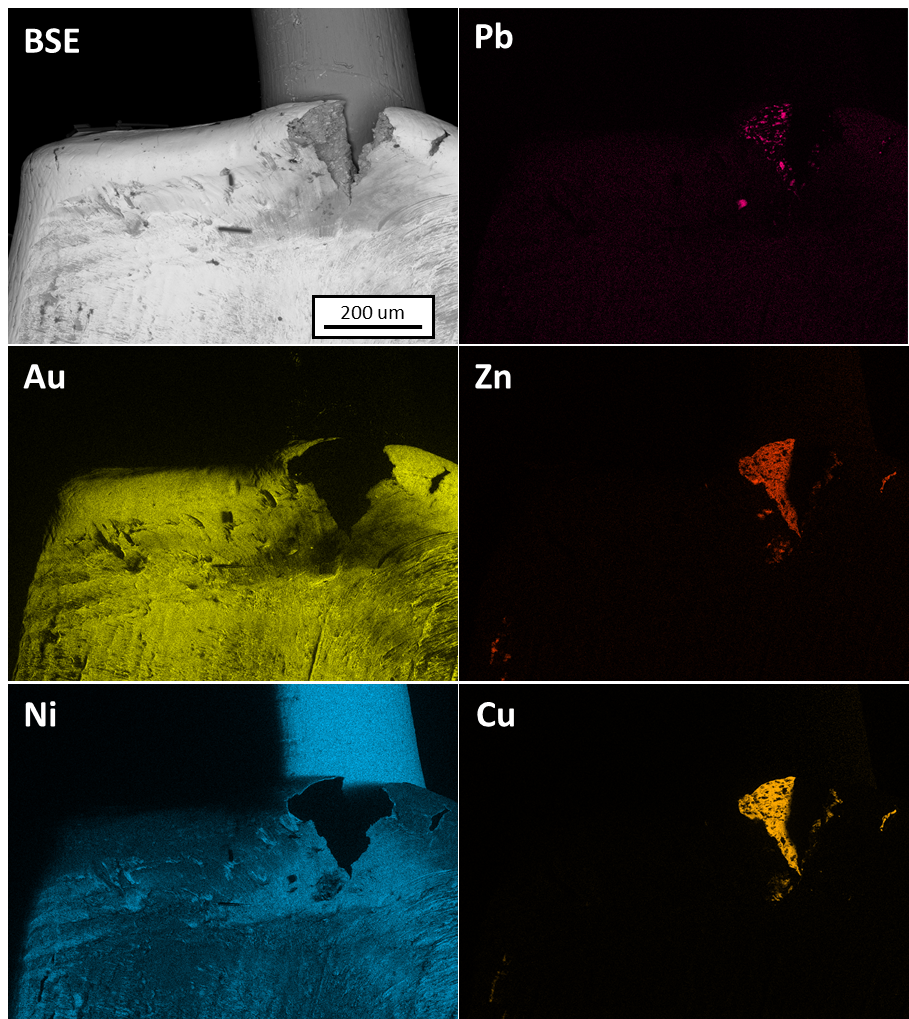


**Figure S2.** SEM-EDS backscatter electron (BSE) and chemical intensity maps of Sample D Site 2

**References**

1 United States Pharmacopeial Convention. (2020). Elemental impurities—Limits (USP <232>). In United States Pharmacopeia 43–National Formulary 38. Rockville, MD: United States Pharmacopeial Convention
